# Rhizodeposition Drives One Third of Belowground Carbon Input in Agroecosystems

**DOI:** 10.1002/advs.78040

**Published:** 2026-09-29

**Authors:** Zhengjun Yan, Yuan Wen, Jie Zhou, Yakov Kuzyakov, Damien Beillouin, Johannes Lehmann, Yadong Yang, Yiqi Luo, Pete Smith, Zhaohai Zeng, Huadong Zang

**Affiliations:** ^1^ State Key Laboratory of Maize Bio‐Breeding College of Agronomy and Biotechnology China Agricultural University Beijing China; ^2^ College of Agronomy Anhui Agricultural University Hefei China; ^3^ Soil and Crop Sciences Section School of Integrative Plant Science Cornell University Ithaca New York USA; ^4^ College of Agriculture Nanjing Agricultural University Nanjing China; ^5^ Department of Soil Science of Temperate Ecosystems Department of Agricultural Soil Science Georg August University of Göttingen Göttingen Germany; ^6^ Peoples Friendship University of Russia (RUDN University) Moscow Russia; ^7^ CIRAD UPR HORTSYS Montpellier France; ^8^ Institute of Biological and Environmental Sciences University of Aberdeen Aberdeen UK

**Keywords:** belowground carbon input, carbon cycling, isotope labeling, plant carbon allocation, rhizodeposition

## Abstract

Soil carbon (C) accumulation, an important mitigation solution to climate change, critically depends on plant C inputs. However, the allocation of belowground C inputs between root biomass and labile rhizodeposits remains poorly quantified. By synthesizing 746 global observations using ^13^C/^14^C tracing, we reveal that 17% of plant assimilated C is allocated belowground, with rhizodeposition accounting for one‐third of the net belowground C input. Legumes primarily allocate assimilated C to belowground via rhizodeposition, whereas other crops, including barley, wheat, rice, maize, and grasses, invest more in root biomass. Rhizodeposition increases with soil organic C up to a peak at 16–19 g C kg^−^^1^ (reaching 8%–10% of assimilated C) and declines thereafter, reflecting a nonlinear relationship between soil fertility and belowground C investment. Environmental change modifies these dynamics as warming suppresses belowground C input by 36%, while drought increases rhizodeposition by 21%. Globally, wheat, maize, and rice contribute 2.70 ± 1.40, 1.76 ± 1.39, and 2.38 ± 1.78 Tg C year^−^^1^ to belowground net C input, respectively. These findings fill critical knowledge gaps in the global C cycle and establish crop‐specific belowground C partitioning coefficients, providing updated empirical parameters to improve process‐based soil C modeling and precise C accounting for climate‐smart agriculture.

## Graphical abstract

This study quantifies photosynthesized C allocation in the plant–soil system across six crop types: barley, wheat, maize, rice, legumes and grasses. It reports C partitioning into shoot respiration, shoot‐C, root‐C, net rhizodeposition and root‐derived CO_2_, providing standardized percentage averages and GPP‐derived absolute values as a robust baseline for agroecosystem C cycling research.

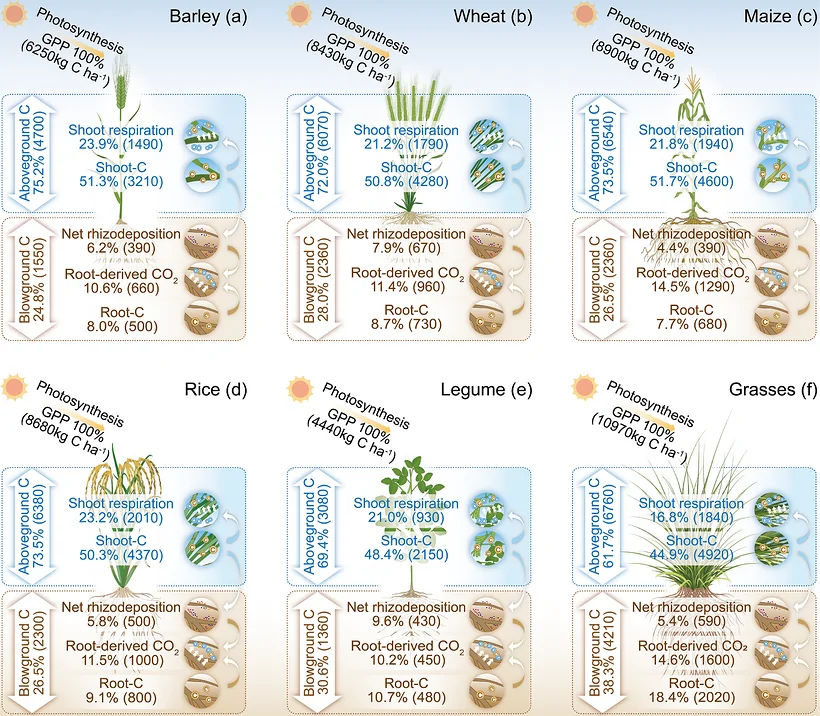

## 1 Introduction

As the largest terrestrial carbon (C) reservoir, soils play a crucial role in regulating global climate by serving as a C sink and buffering atmospheric CO_2_ emissions [1, 2]. Nearly all soil organic C (SOC) originates from plant residues, with belowground C input—particularly root residues and rhizodeposition—being 2.4 times more efficiently stabilized as SOC than aboveground residues [3, 4]. This belowground stabilization efficiency fundamentally depends on the ‘dead‐or‐alive’ dichotomy: ‘dead’ inputs (root residues) versus ‘alive’ inputs (rhizodeposits) [5, 6]. Rhizodeposition exhibits a sixfold higher efficiency than root residues in forming mineral‐associated organic matter, highlighting its contribution to long‐term soil C persistence [7]. Nevertheless, precise quantification of rhizodeposition remains experimentally challenging, introducing considerable uncertainty into existing estimates [8]. These knowledge gaps limit our understanding of soil C dynamics and hinder the development of effective strategies for enhancing soil C accumulation in agroecosystems [9].

Fundamentally, the dynamics of belowground C input depend on how plants allocate photosynthetic C between roots and rhizodeposits. This allocation is highly variable and shaped by multiple biotic and abiotic factors [10, 11], including plant traits [12], soil properties [13, 14], and climate [15]. Plant traits such as plant genotype, root morphology, and rhizodeposit composition govern the quantity and quality of belowground C inputs [2], while plant ontogeny introduces temporal variation as allocation patterns shift across developmental stages [16, 17]. Soil characteristics—SOC content, nutrient availability, and pH—modulate plant growth and biomass production, which in turn affect the photosynthetic C allocation within the plant [18]. Climate, particularly temperature and precipitation, further shapes belowground C allocation by influencing photosynthesis, plant respiration, root growth, and resource allocation to roots and rhizodeposits [19, 20]. Environmental change further amplifies this complexity, with elevated CO_2_, warming, and drought introducing additional uncertainty [10, 21, 22, 23]. Despite the critical role of belowground C allocation, a comprehensive understanding of its global patterns and the key drivers remains limited, especially in agroecosystems.

Here, we compiled and analyzed the largest globally standardized dataset of 746 observations spanning major crop types and climate zones (Figure S1). Using machine learning algorithms, we aimed to: (1) quantify the allocation of belowground C inputs between root biomass and labile rhizodeposits; (2) identify the key biophysical and climatic drivers of belowground C input; and (3) generate spatial estimates of belowground C input for global major crops to provide improved baseline parameters for SOC models and agricultural C credit frameworks.

## 2 Results and Discussion

### 2.1 More Than One Third of Belowground Net C Input Occurs Via Rhizodeposition

Our systematic review found that the assimilated C was predominantly allocated aboveground, with 49% stored in shoot biomass, 34% lost through shoot and root respiration, and only 17% retained as belowground net C input. Within this belowground component, over one‐third (35%) of the net input is allocated to rhizodeposits, while the remainder contributes to root biomass C (Figure 1 and Figure S2). To assess methodological uncertainty, we further grouped all observations by root separation protocols following Liang et al. [24]. While absolute rhizodeposition estimates varied across methods, the relative ratio of rhizodeposition to net belowground C input (ranging from 32% to 38%) was not substantially affected by the separation methods (Figure S3). This quantitative partitioning offers a comprehensive global perspective on belowground C input across diverse climate zones and ecosystems, extending beyond previous regional and crop‐specific analyses.

**FIGURE 1:**
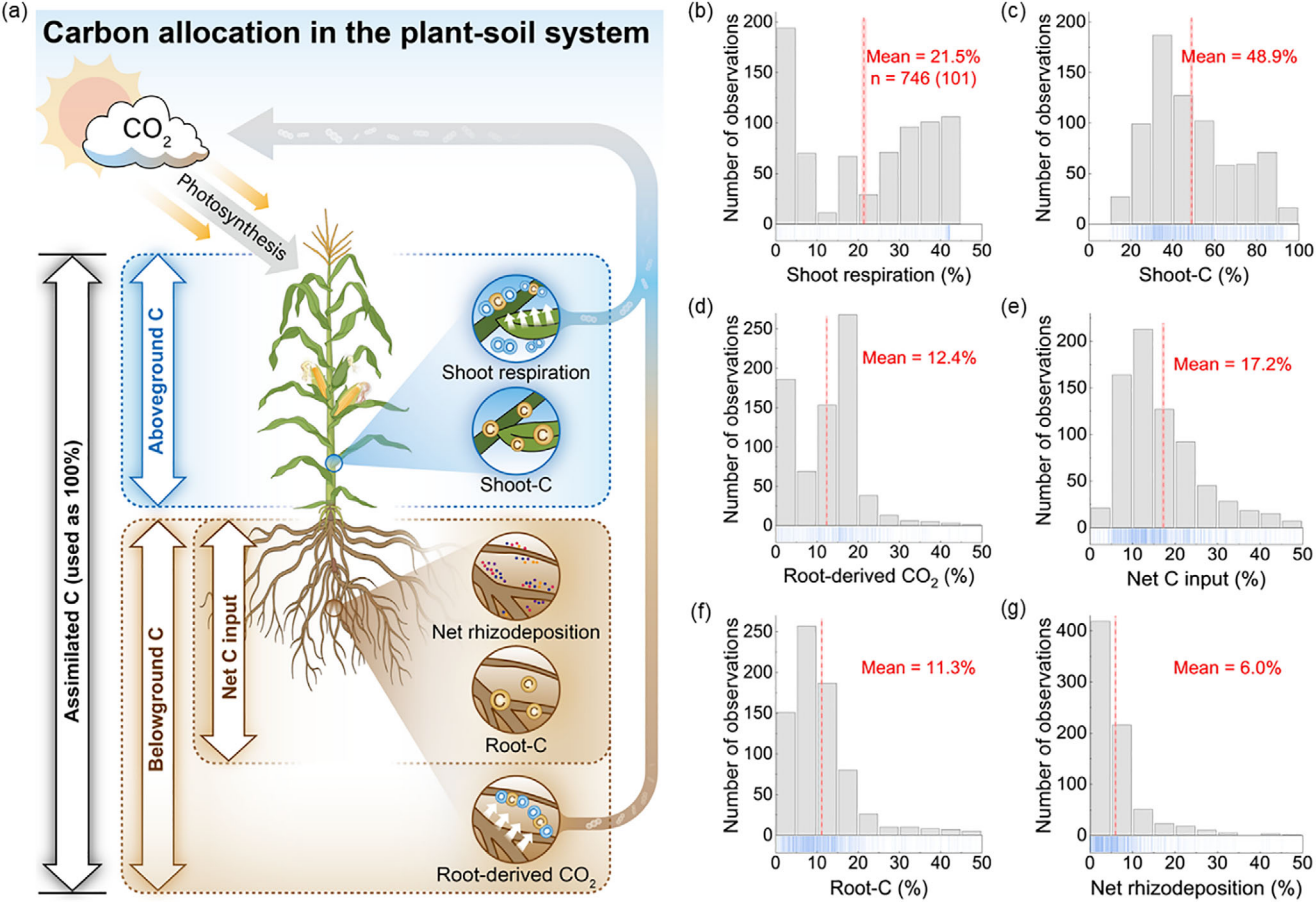
Photosynthetic carbon (C) allocation in the plant‐soil system and its quantification across various pools. A conceptual diagram illustrating the fate of photosynthetic C within the plant‐soil system (a). Arrows represent the fluxes between pools. Aboveground C and belowground C refer to the amounts of C allocated to the respective pools. The net C input is defined as the sum of rhizodeposition and root‐C. Assimilated C represents the total amount of C assimilated by plants, encompassing both retained C (in plant and soil pools) and C released via fluxes (e.g., respiration). All values reported in the literature were standardized relative to assimilated C (set to 100%). See Methods for details on data harmonization. Distribution of C allocation to key pools and fluxes: shoot respiration (b), shoot‐C (c), root‐derived CO_2_ (d), net C input (e), net rhizodeposition (f), and root‐C (g). The red dashed lines and the red shaded area indicate the means and the standard errors. The blue solid lines along the X‐axis represent the distribution of observations within each C pool.

Distinct crop‐specific C allocation patterns between root and rhizodeposits were observed, with root‐dominant patterns occurring 2.7 times more frequently than rhizodeposition‐dominant patterns (548 vs. 198; Figure 2). Grasses showed the highest root‐C allocation proportion, likely reflecting adaptation to resource‐limited environments, such as nutrient‐poor soils or arid regions. In such contexts, deeper or more extensive root systems benefit nutrient and water uptake, thereby improving plant resilience [25, 26, 27]. However, this root‐dominant allocation was not restricted to grasses or resource‐limited environments. Even maize and rice—typically cultivated in water‐ and nutrient‐sufficient environments—allocated a greater proportion of their belowground C to root rather than rhizodeposition (Figure 2). Such a pattern may reflect long‐term selection for genotypes with rapid root development and efficient resource acquisition [28, 29]. In contrast, legumes allocated a greater proportion of assimilated C to rhizodeposition than to root biomass (Figure 2). This pattern appears linked to their extensive root branching and dense root hair systems, which increase rhizosphere interactions and rhizodeposition accumulation [30, 31]. These root traits support nitrogen fixation, soil nutrient mobilization, and beneficial microbial recruitment. As highlighted by Freschet et al. [32], root trait spectra such as lifespan, tissue density, and exudation potential mediate not only nutrient acquisition but also C residence time in soils. Integrating this trait‐based understanding can improve predictions of C stabilization across agroecosystems, and refine management practices that increase soil health and sustainability.

**FIGURE 2:**
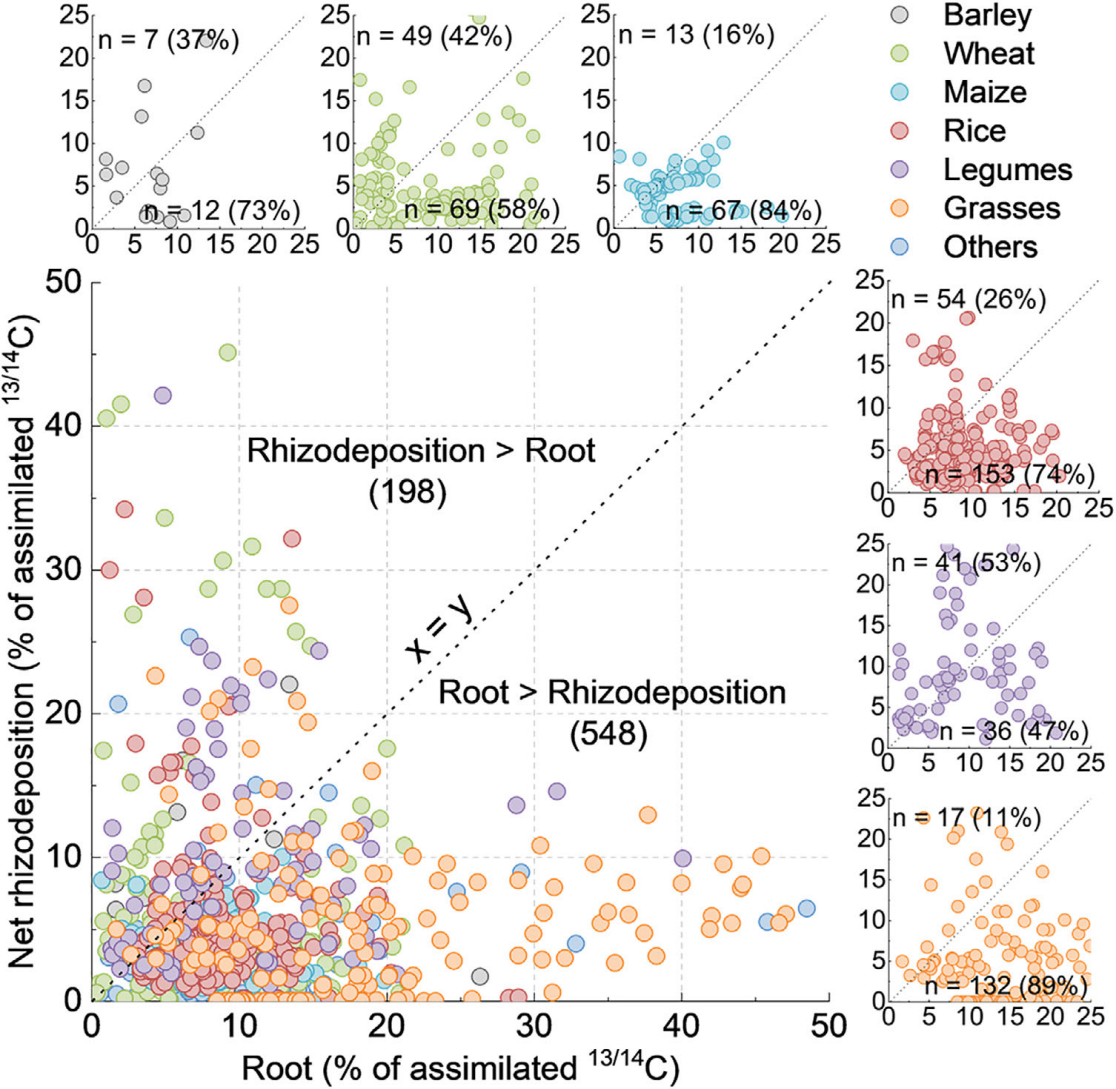
Distribution of photosynthesized C allocated to roots and rhizodeposition. The figure shows the proportion of assimilated ^13/14^C allocated to roots and rhizodeposition across the full dataset and for individual crops (barley, wheat, maize, rice, legumes, and grasses). For visualization purposes, the upper limit of both axes was capped at 25% for each crop; data points exceeding this threshold are not visible in the plots. Numbers in parentheses represent the number of points above and below the 1:1 line, respectively.

### 2.2 Higher Rhizodeposition and Belowground Net C Input at Moderate SOC Contents

On average, rhizodeposition and belowground net C input increased with initial SOC content, followed by a decline beyond a critical threshold (Figure 3). The optimal SOC range was 17.1–18.5 g kg^−^^1^ for rhizodeposition and 16.4–17.1 g kg^−^^1^ for net C input. At these optimal SOC levels, plants allocated 8.2%–9.6% of assimilated C to rhizodeposition and 19.1%–20.3% to net C input (Figure 3). At low SOC levels, nutrient limitation often constrains plant growth and total photosynthetic C assimilation, which in turn limits root exudation and belowground C allocation [14, 33]. As SOC increases to moderate levels, nutrient availability and microbial interactions improve, promoting greater belowground C partitioning to roots and rhizodeposits [34, 35]. However, under high SOC conditions, further increases in nutrient availability may reduce the need for lateral root development and rhizodeposition [36]. These findings reveal the nonlinear relationship between soil properties and belowground C allocation, suggesting a threshold beyond which resource saturation may suppress plant investment in the rhizosphere. These results also support frameworks in which root plasticity and C allocation patterns respond dynamically to the local nutrient context [32, 34, 36].

**FIGURE 3:**
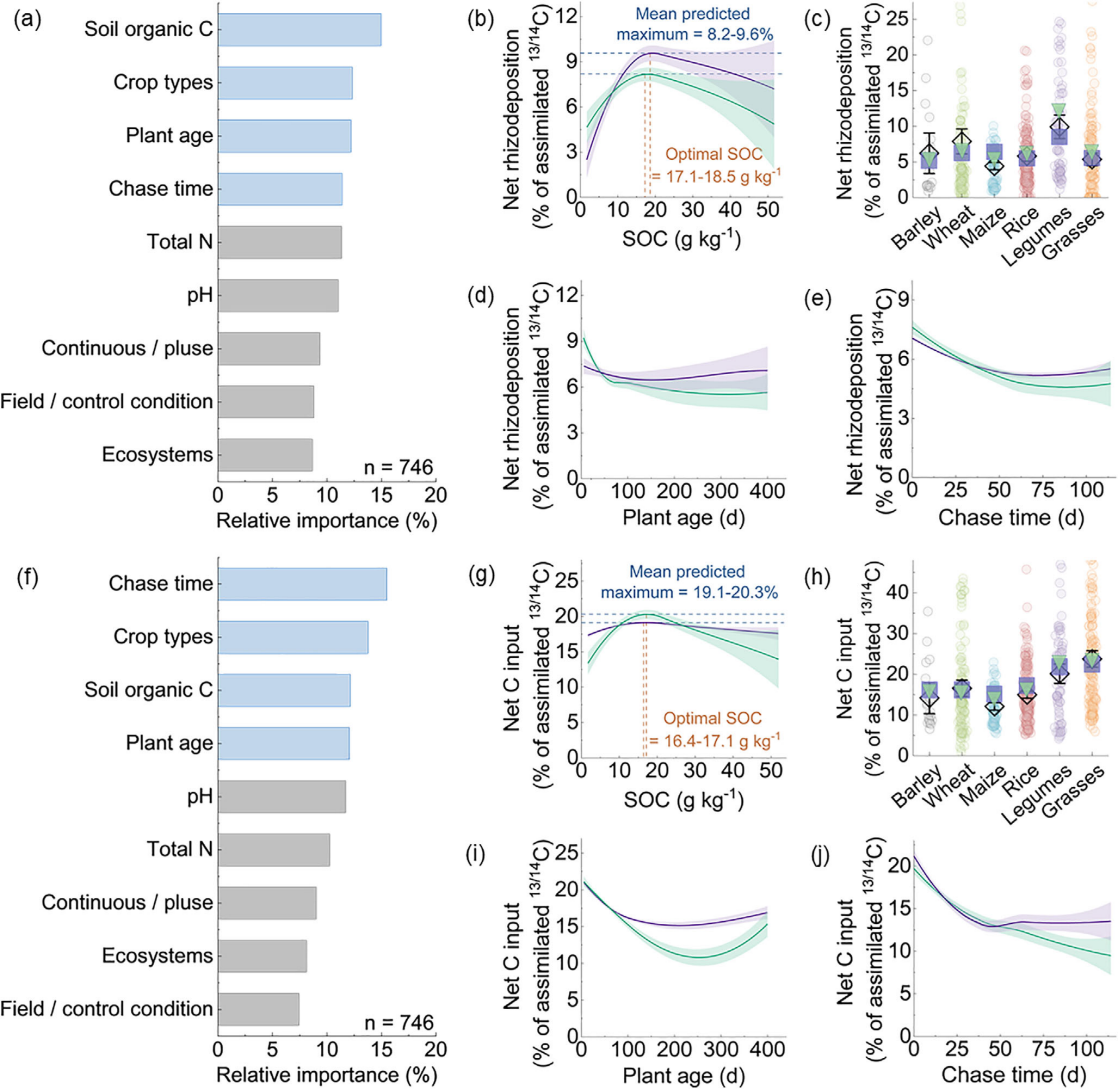
Factors determining belowground C input by crops. The relative importance of the variables in explaining the net rhizodeposition (a) and net C input (f) is based on the mean value across both the random forest and gradient boosting machine models, using RMSE loss after permutation. The detailed model performance is provided in Table S3. Partial dependence plots show the effect of soil organic C on rhizodeposition (b) and net C input (g), with predictions from random forest (purple) and gradient boosting machine (green) analyses. The SOC range indicates the SOC content maximizing C allocation belowground. Boxplots display the data distribution of net rhizodeposition (c) and net C input (h) across six crop types (barley, wheat, maize, rice, legumes, and grasses). The diamonds with error bars indicate mean values with 95% confidence intervals, while green triangles and purple squares refer to the mean predictions from gradient boosting machine and random forest analyses, respectively. Partial dependence plots show the influence of plant age (d, i) and chase time (the duration between isotopic labeling and sampling) (e, j) on net rhizodeposition and net C input. Shaded areas represent 95% confidence intervals.

### 2.3 Legumes Allocate Highest Proportion of Belowground Net C to Rhizodeposition

Among the six crop types examined, legumes allocated the highest proportion of assimilated C to rhizodeposits (9.9% ± 1.6%), consistent with their overall tendency to favor rhizodeposition over root biomass allocation (Figure 2). This represents a 30%–120% increase compared to other crop groups (Figure 3c). This might be attributed to their ability to perform biological nitrogen fixation, which requires substantial C investment in nodule formation and maintenance, thereby increasing the flux of rhizodeposition [37]. Additionally, legumes typically develop extensive lateral root systems and dense root hairs that enhance rhizosphere interactions and promote rhizodeposition [32, 38]. In contrast to the rhizodeposition‐dominated strategy of legumes, grasses showed the highest overall belowground net C input as 23.8% ± 2.0% of total assimilated C (Figure 3h). This pattern can be attributed to their extensive root systems, which optimize resource acquisition and distribution, and their reliance on substantial C reserves in roots for spring regeneration and recovery after grazing or mowing [39, 40]. The higher root‐to‐shoot ratio in grasses compared to annual crops confirms a greater C allocation to roots. Maize had the lowest levels of both net rhizodeposition (4.4% ± 0.5%) and net C input (12.1% ± 0.9%) among the six crop types (Figure 3c–h). This was likely because a larger proportion of photosynthetic C was allocated to aboveground biomass and grain rather than belowground C input [41]. Such contrasting allocation patterns among crop types may reflect differences in root economic strategies [42], with legumes exhibiting acquisitive root traits (e.g., high exudation, low tissue density) and grasses showing conservative traits (e.g., dense, long‐lived roots). This trait‐based distinction can inform the selection of complementary crop combinations—for example, pairing legumes with grasses—to simultaneously enhance nutrient cycling, improve soil structure, and increase long‐term C stabilization in agroecosystems. Notably, this is being applied in grass‐clover systems, which are widely used worldwide for both grazing and forage production.

### 2.4 Temporal Shift in Belowground C Allocation

Rhizodeposition and belowground net C input initially declined with increasing crop age, reaching their lowest levels at approximately 60 and 120 days after planting, respectively, before stabilizing (Figure 3d–i). This aligns with the well‐established plant C allocation strategy, in which plants have a higher root‐to‐shoot ratio during early rapid vegetative growth compared to more mature stages [43]. In juvenile plants, more C was allocated to root development for nutrient uptake, while mature plants prioritized shoot growth for photosynthesis and reproduction [44, 45]. This age‐related decline in belowground allocation was particularly pronounced in annual crops such as wheat, maize, and soybean, where root growth slowed or even ceased in the later development stages, resulting in a relative decrease in belowground C input [46]. In contrast, perennial grasses showed relatively stable belowground C allocation after about 200 days. This stability arises from coupled development of shoot and root systems after establishment [40], as well as shifts in C allocation to roots post‐grazing or mowing [47]. Together, these processes likely contribute to the relatively stable belowground C allocation observed in perennial grasses compared with annual crops. These findings highlight how developmental stage constrains belowground C allocation strategies, in line with allometric principles of biomass partitioning [48]. This reinforces the need to consider temporal dynamics when designing sampling protocols or modeling plant‐soil C fluxes.

In addition to plant age, the duration between isotopic labeling and sampling—referred to as chase time—also influenced belowground C fluxes. Both rhizodeposition and net C input decreased with increasing chase time, stabilizing after approximately 40 days (Figure 3e–j). This trend can be explained by the distinct turnover of fine roots and rhizodeposits. Labile rhizodeposits have much faster turnover and are rapidly decomposed into CO_2_ by soil microbes in the short term [10, 49, 50]. Crucially, this decline is driven primarily by microbial CO_2_ release rather than by C translocation from roots to shoots [51], as such root‐to‐shoot C transfer occurs only in the late reproductive stages of annual crops and therefore cannot explain this general trend [52]. As chase time increased, net C input became more stable, indicating a dynamic balance of the continuous C inputs from plants and outputs via microbial mineralization [53]. These findings underscore the dynamic interplay between belowground C input and time, crucial for comprehending soil C accumulation.

### 2.5 Rhizodeposition and Belowground C Partitioning Under Environmental Change

Our synthesis revealed contrasting responses of belowground C dynamics to key environmental change drivers (Figure 4). Experimental warming significantly reduced both rhizodeposition and belowground net C input by 70.7% and 35.5%, respectively, compared to the non‐warming condition (Figure 4b). These findings align with Terrer et al. [54], who showed that warming weakens plant‐soil feedbacks by altering mycorrhizal associations and reducing root exudation, thereby limiting soil C accrual. This decline was driven by two countervailing mechanisms: while elevated temperatures increased photosynthetic C assimilation, they also increased respiratory C losses from both shoots and roots, and suppressed root biomass [55]. In contrast, drought increased rhizodeposition by 21.2% (Figure 4a)—a response consistent with plants prioritizing root exudation to increase water acquisition through microbial symbiosis [56] and soil structure stabilization [57]. In addition, nitrogen addition exerted consistent negative effects (Figure 4), decreasing both net rhizodeposition (‐19.6%) and belowground net C input (−17.5%). These declines aligned with optimal partitioning theory [58], as nutrient enrichment reduced the demand for root foraging, favoring allocation to shoot over root [59, 60]. These results have important implications for agroecosystems in regions of intensive N application, where increased aboveground productivity may be accompanied by diminished belowground soil C accrual potential [61]. Collectively, these findings underscore the intricate and context‐dependent responses of belowground C dynamics to environmental drivers. Effectively managing these belowground processes—especially under warming, more frequent droughts, and nutrient enrichment—will be essential for sustaining soil C sinks and enhancing agroecosystem resilience under global change [54, 62, 63].

**FIGURE 4:**
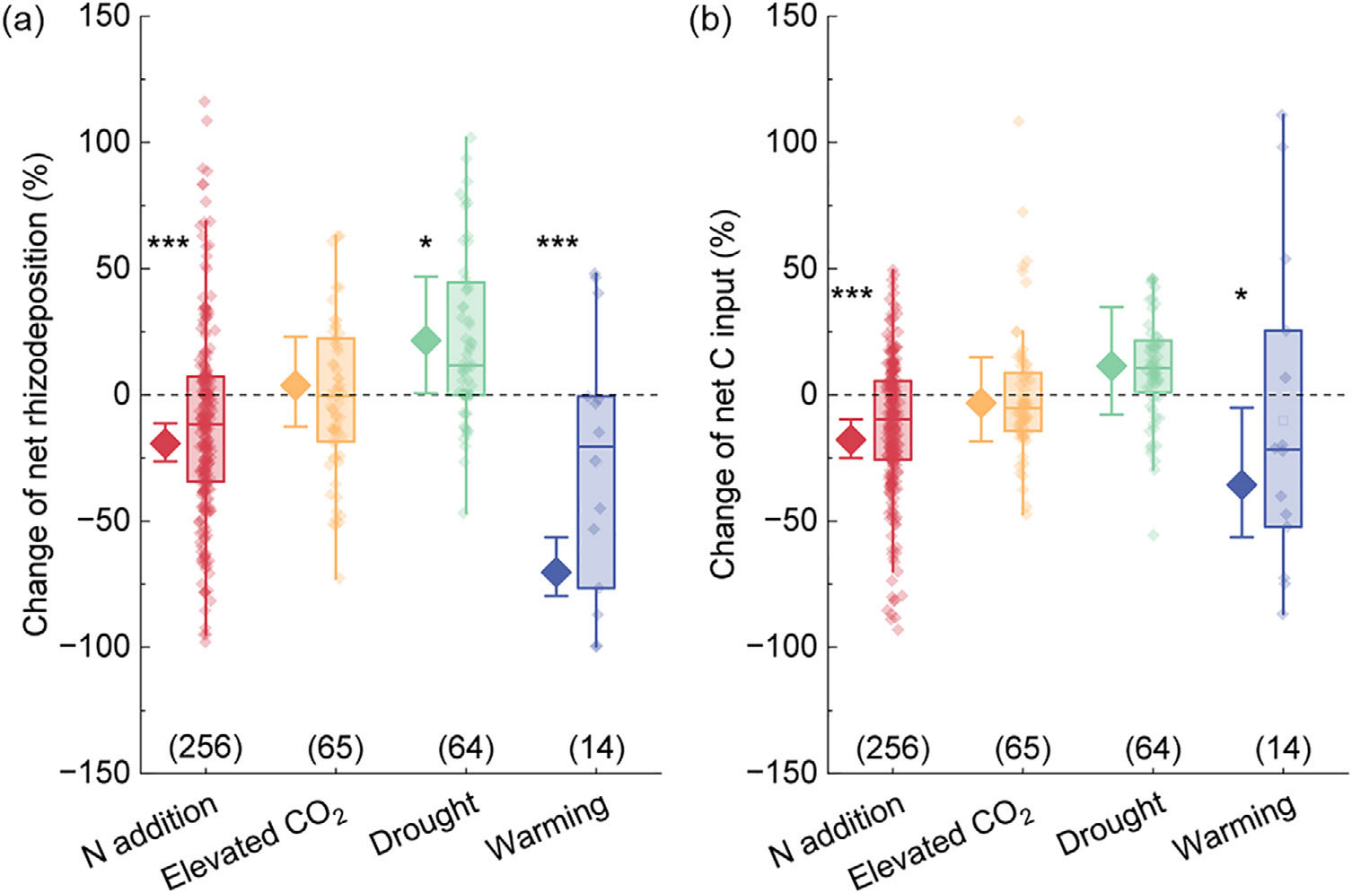
Belowground C input under key environmental change factors. Relative changes (%) of net rhizodeposition and net C input as affected by nitrogen (N) addition, elevated CO_2_, drought, and warming. Diamonds with vertical bars indicate the mean effect (±95% CI). Small points show individual paired contrasts; central box plots show the median and interquartile range (IQR), with whiskers extending to 1.5×IQR. Asterisks denote two‐sided tests of the mean effect against zero (^*^ *p* < 0.05, ^**^ *p* < 0.01, ^***^ *p* < 0.001). The numbers in parentheses refer to the number of pairwise observations. All effects are derived from ^13/14^C labeling studies; see Methods in Supporting Information for data analysis details.

### 2.6 Global Distribution of Belowground C Input and Policy Pathways for Soil C Stewardship

Globally, the average belowground net C input for wheat, maize, and rice was estimated at 1.76 ± 0.03, 1.18 ± 0.02, and 1.85 ± 0.03 Mg C ha^−1^ year^−1^, respectively (Figure 5). In absolute terms, this translates to total global belowground net C input of 2.70 ± 1.40, 1.76 ± 1.39, and 2.38 ± 1.78 Tg C year^−1^ for wheat, maize, and rice, respectively (Figure 5; Table S1). These estimates were calculated using crop‐specific belowground net C input proportions and rhizodeposition‐to‐root ratios derived from this study (Figure S4). The average rhizodeposition‐to‐root ratio across all crops in our dataset was 0.63, closely matching the widely adopted general coefficient of 0.65 reported by Bolinder et al. [64]. However, the large crop‐specific divergence we uncovered (0.26–0.97) highlights critical limitations of uniform constant parameters in existing C models. Incorporating our crop‐differentiated partitioning values will markedly improve the precision of cropland C flux simulations and national greenhouse gas inventories.

**FIGURE 5:**
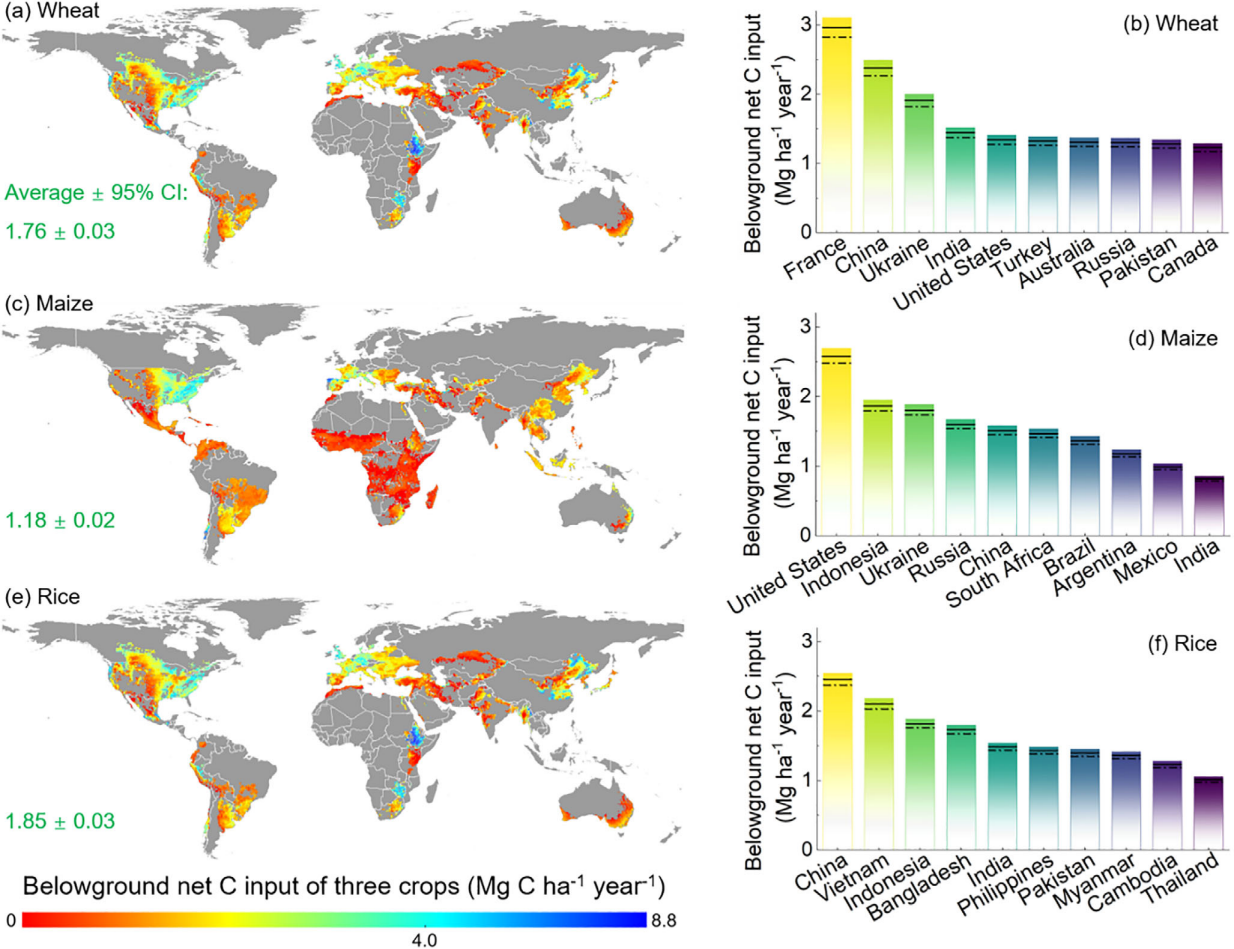
Global belowground net C input. Spatial patterns of annual belowground net C input are shown for wheat (a), maize (c), and rice (e). Gray areas indicate regions with no available data. The global yield data for the three crops (wheat, maize, and rice) were obtained from [65]. The generalized percentage values of net C input for the three crops were calculated as averages of all collected data for each crop, representing percentages of the gross primary production (GPP). According to the relationship between crop yield and GPP based on [64], we mapped the global spatial distribution of belowground net C input using QGIS version 3.32 (Open Source Geospatial Foundation Project, http://qgis.osgeo.org). The global map was obtained from Natural Earth (http://www.naturalearthdata.com/). Panels b, d, and f show estimates of belowground net C input for the ten highest‐producing countries for wheat, maize, and rice, respectively, based on the most recent FAO production statistics (2023). Bars indicate maximum values; black dashed and solid lines represent minimum and mean values, respectively. Extended data for the top 50 producing countries are available in Table S1.

Widely used soil C models, C inventories and emerging agricultural C credit schemes currently rely on a single generalized rhizodeposition‐to‐root coefficient, thereby overlooking substantial functional differences among crops [66, 67]. This oversimplification introduces considerable uncertainty in regional and national cropland C sink estimates. Our crop‐differentiated rhizodeposition and root C partitioning ratios, paired with global spatial distribution data, enable refined model parameterization and regionally differentiated C accounting. Our finding of an optimal SOC range of approximately 16–19 g kg^−1^ for maximizing belowground C input—beyond which belowground C input declines by 8%–74%—provides a quantitative factor for regionally targeted soil management policies. Rather than offering direct agronomic guidance, our spatial datasets provide quantitative baselines to support targeted C sink policy design and reliable C credit quantification.

Furthermore, by revealing that over one third of belowground C input originates from rhizodeposition, this work bridges theoretical C allocation frameworks with the quantitative data urgently needed for model refinement. It reinforces the need to integrate rhizosphere processes into C accounting frameworks. By making this major, yet often overlooked, C pathway measurable and amenable to model parameterization, our findings support science‐based progress toward Sustainable Development Goals (SDGs) 2 (Zero Hunger) and 13 (Climate Action), thereby balancing agricultural productivity with planetary health.

## 3 Methods

### 3.1 Study Selection

We conducted a comprehensive literature search in both English and Chinese databases: the Web of Science (http://www.webofknowledge.com) and the China National Knowledge Infrastructure (https://www.cnki.net). We focused on studies that quantitatively assessed belowground C input of crops utilizing stable (^13^C) or radioactive (^14^C) isotope labeling techniques. The search strings (Supplementary Table 2) yielded 163 studies published from 1986 to 2024. Additionally, we complemented this search with a backward citation search, screening the reference lists of selected papers from previous reviews [22, 39, 68, 69, 70]. The literature search followed the PRISMA (Preferred Reporting Items for Systematic Reviews and Meta‐Analyses) flow diagram (Figure S5).

Studies from the retrieved publications were screened using the following inclusion criteria: (1) the study employed either pulse or continuous labeling with ^13^CO_2_ or ^14^CO_2_; (2) the partitioning of assimilated C in the plant‐soil system was reported directly as numerical, graphical data, or could be calculated from the reported data; (3) the partitioning of assimilated C included at least root and rhizodeposition pools; (4) the crop type, plant age, and chase time were provided. When multiple publications reported results from the same experiment setup, these were consolidated and treated as a single study. Finally, 746 observations from 101 publications were identified as suitable for further analyses. The list of data sources is provided as Supplementary Information.

### 3.2 Data Extraction

Data on assimilated C partitioning were reported in various formats: (1) ^14^C activities or ^13^C amounts in individual pools; (2) total labeled C introduced into the plant‐soil system; (3) total assimilated C; or (4) C recovery at a specific time after labeling. We extracted numerical values, replication numbers, and the standard deviation of the C allocation in each pool. In cases where data were only presented in figures, values were extracted using the GetData Graph Digitizer (http://getdatagraphdigitizer.com/). For each study, we recorded the following variables: (1) plant and soil characteristics: soil properties (pH, C and N contents, and C/N ratio), shoot and root biomass, root‐to‐shoot ratio, ecosystem type (cropland/grassland); (2) experimental design: labeling methods (pulse or continuous), field or controlled conditions, chase time, and plant age; (3) C allocation metrics: shoot respiration, shoot C, root C, rhizodeposition C, and root‐derived CO_2_; (4) climatic variables: mean annual temperature (MAT) and mean annual precipitation (MAP), either reported in the study, or derived from the WorldClim database using reported geographic coordinates. Here, rhizodeposition C refers to net rhizodeposition—the fraction retained in soil after microbial mineralization—and thus provides a conservative lower‐bound estimate of total root‐derived C inputs. In addition, we classified observations into six crop types: barley, wheat, maize, rice, legumes, and grasses. Barley, wheat, maize, and rice were treated as individual crop species, whereas legumes and grasses were treated as broader taxonomic/functional categories comprising multiple species. Observations that could not be assigned to any of these six types were pooled into an “Others” category and excluded from the six‐type comparisons. This classification was applied consistently throughout the analyses.

### 3.3 Data Standardization

To ensure cross‐study comparability, we first standardized C partitioning values by recalculating them as a percentage of total assimilated C (Figure 1a). This normalization step was essential since some studies do not account for ^13^CO_2_ or ^14^CO_2_ losses via shoot respiration and/or root‐derived CO_2_ when reporting C budgets, leading to potential overestimation of remaining C pools and bias in inter‐study comparisons. To address this, we modeled the shoot respiration and root‐derived CO_2_ as functions of chase time (Figure S6), and used these models to estimate missing data. Due to limited data availability per crop, two generic curves were fitted across all vegetation types. While this approach necessarily smooths over some species‐specific physiological variation, it provides a consistent and necessary correction for cross‐study comparison, notably reducing bias compared to ignoring these fluxes entirely. The resulting dataset was thus fully expressed as a percentage of assimilated C, ensuring comparability with photoassimilate studies in plant ecophysiology.

### 3.4 Carbon Partitioning and Their Drivers

We applied a machine‐learning‐based meta‐analytic framework to predict belowground net C input and rhizodeposition. Following Zhao et al. [71], we implemented *metaforest* in R, beginning with a broad set of 12 moderators, and retaining only those without missing values and with predictive significance (Figure S7). Moderator importance values were calculated based on the Gini impurity index, which quantifies each variable's importance to improving node purity across all decision trees. If the variable was useful, it tended to split mixed‐label nodes into pure, single‐class nodes.

Specifically, moderator pre‐selection was conducted via the *preselect* function in the metaforest package, using the recursive algorithm based on 10 000 iterations and 100 replications. Moderators with consistently relatively low predictive values and extensive missing values were excluded. Then, the tuning parameters of *metaforest* were optimized using 10‐fold cross‐validation to mitigate overfitting via the *train* function from the *caret* package, minimizing the root‐mean‐square error (RMSE). The final metaforest model included 746 observations with 9 selected predictors.

Subsequently, we trained gradient boosting machine (GBM) and random forest (RF) to independently predict belowground net C input and rhizodeposition. The top four most influential variables—soil organic C, crop types, plant age, and chase time—determined through a permutation method [72] were selected for further interpretation via partial dependence analysis, implemented in the “DALEX” package (Figure S8). This allowed us to estimate the mean causal effect of these variables on belowground net C input and rhizodeposition, while accounting for variable interactions. Smoothed response curves were fitted using locally weighted regression (*loess)*, providing a clear understanding of trends and changes in the data. The 95% confidence intervals were determined using non‐parametric bootstrapping with 1000 replicates. Associated scatter plots are shown in Figure S9.

### 3.5 Key Environmental Change Effects

An in‐depth analysis was conducted to determine the impact of global change factors—such as nitrogen (N) addition, elevated CO_2_, drought, and warming—on belowground net rhizodeposition and net C input based on the subset. Our dataset included 256 paired observations for N addition, 65 for elevated CO_2_, 64 for drought, and 14 for warming. To quantitatively assess the effects of these global change factors on belowground C input, we employed the natural log‐transformed response ratio (lnRR) as follows:

(1) $$In\;RR=In\left({\bar{X}}_{t}/{\bar{X}}_{c}\right)=In\left({\bar{X}}_{t}\right)-In\left({\bar{X}}_{c}\right)$$

where $\bar{X_{t}}$ represents the mean values of the treatment plots under global change factors, and $\bar{X_{c}}$ represents the mean values at control plots. In our database, standard deviations were missing in most studies, but the sample sizes were available. The weight (*W*) for each lnRR observation was calculated using the equation as follows [22]:

(2) $$W_{i}=\left(N_{t}\times N_{c}\right)/\left(N_{c}+N_{t}\right)$$

where *W_i_* is the weight for each lnRR observation, and *N_t_* and *N_c_* are the number of replicates in the treatment and control groups, respectively. This approach allowed us to account for the variability and reliability of the data when assessing the effects of global change factors on belowground C input. The weighted effect sizes were calculated as follows:

(3) $$In\;RR_{++}=\sum \left(InR_{i}\times W_{i}\right)/\sum W_{i}$$

where lnR*_i_* and *W_i_* are the effect size and weight of corresponding comparisons. Therefore, the 95% CI of lnRR_++_ was computed as per Equation (4):

(4) $$95\%Cl=In\;RR_{++}\pm 1.96S_{InRR_{++}}$$

where *S*_lnRR++_ is the SE of lnRR_++_, which was calculated as follows:

(5) $$S_{InRR++}=\sqrt{1/\sum W_{i}}$$

To enhance readability and interpretation, the lnRR_++_ ± 95% CI was transformed back to the percentage change as (e^lnRR++^ – 1) × 100% for ease of interpretation. The effects of the key environmental change factors were considered significant if the 95% CI did not overlap zero.

### 3.6 Estimation of Global Absolute Carbon Fluxes

To quantify the absolute values for shoot respiration, shoot‐C, root‐C, net rhizodeposition, and root‐derived CO_2_ for various crops, we combined C‐partitioning results from this review with estimated gross primary production (GPP) or net primary production (NPP) drawn from the literature [39, 64, 73]. GPP is the total assimilated C by photosynthesis, while NPP is the remainder after autotrophic respiration. The C‐partitioning percentages obtained in this review were averaged across crop types and expressed as the percentage of GPP. NPP was generally calculated based on the equations as follows [64]:

(6) $$NPP=C_{P}+C_{S}+C_{R}+C_{E}$$

(7) $$C_{P}=Y_{p}\times 0.45$$

(8) $$C_{S}=Y_{P}\left(1-HI\right)/HI\times 0.45$$

(9) $$C_{R}=Y_{P}/\left(S:R\times HI\right)\times 0.45$$

(10) $$C_{E}=C_{R}\times Y_{E}$$

where *C_P_* is the plant C in the agricultural product, the plant portion of primary economic value; *C_S_* refers to the plant C in aboveground non‐grain biomass, including stems, leaves, and other post‐harvest residues; *C_R_* represents the plant C in root tissue; *C_E_* indicates plant C in extra‐root material, including root exudates and other material derived from root turnover; *Y_P_* is the dry matter yield of aboveground products (kg ha^−1^ year^−1^), which is derived from the latest production statistics (2023) provided by the Food and Agriculture Organization (FAO) (https://www.fao.org); the factor 0.45 is the C content of dry biomass in grain crops, a standard conversion coefficient following Bolinder et al. [64]; *HI* refers to the harvest index; *S: R* is the shoot: root ratio, and *Y_E_* represents the ratio of extra‐root C (rhizodeposit C) to recoverable root biomass C, with the values derived from crop‐specific empirical rhizodeposition‐to‐root ratios obtained from our study. We quantified the allocation of photosynthetic C for various crops based on the partitioning results generalized in this review and GPP data obtained from the literature (Figure S4). These generalizations can be applied to estimate absolute belowground C input across various crops, as well as for upscaling from plot to agroecosystem levels.

### 3.7 Spatial Mapping of Belowground C Inputs

Finally, we mapped global spatial patterns of belowground C input for three major staple crops—wheat, maize, and rice—using crop yield data [65] and crop‐specific C allocation ratios derived from this study. Specifically, the generalized percentage values of belowground C input for wheat, maize, and rice represented the proportion of gross primary production (GPP) allocated belowground, derived from averages of comprehensive data collected for each crop type. Utilizing the relationship between crop yield and GPP outlined by Bolinder et al. [64], we employed QGIS version 3.32 (Open Source Geospatial Foundation Project, http://qgis.osgeo.org) to map the global spatial distribution of belowground net C input for these three crops. Furthermore, our analysis included the annual minimum and maximum global belowground net C input values for these three crops, as presented in Figure S10, offering a detailed view of yearly fluctuations in belowground C input.

## Author Contributions

Z.Y. and Y.W. contributed equally to this work. H. Z., Z. Y., and Y. W. conceptualized the study. Z. Y., H. Z., and J. Z. collected data. Z. Y., and H. Z. analyzed data and performed visualization. Y. K., D. B., J. L., and Y. L. provided suggestions for the storylines and data analysis. Z. Z., Y. W and H. Z. received funding and supervised the study. H. Z., Z. Y., and Y. W. wrote the original manuscript and all authors contributed to reviewing and editing the paper.

## Conflicts of Interest

The authors declare no conflicts of interest.

## Supporting information




**Supporting File**: advs78040‐sup‐0001‐SuppMat.docx.

## Data Availability

The data that support the findings of this study are available from the corresponding author upon reasonable request.
