# Supplementary material for: Rhizodeposition Drives One Third of Belowground Carbon Input in Agroecosystems
Source: Adv Sci (Weinh). 2026 Sep 29:e78040. Online ahead of print. doi: 10.1002/advs.78040 (PMC13624308; doi:10.1002/advs.78040)
Supplement: Supplementary file 1 [file ADVS-9999-e78040-s001.docx]

**Supplementary information**

**Title:** Rhizodeposition drives one third of belowground carbon input in agroecosystems

**Author list**

Zhengjun Yan^a,b*^, Yuan Wen^a,c*^, Jie Zhou^d^, Yakov Kuzyakov^e,f^, Damien Beillouin^g^, Johannes Lehmann^c^, Yadong Yang^a^, Yiqi Luo^c^, Pete Smith^h^, Zhaohai Zeng^a^, Huadong Zang^a,c**^

^a^ State Key Laboratory of Maize Bio-breeding, College of Agronomy and Biotechnology, China Agricultural University, Beijing 100193, China

^b^ College of Agronomy, Anhui Agricultural University, Hefei 230036, China

^c^ Soil and Crop Sciences, School of Integrative Plant Science, Cornell University, Ithaca, NY, USA

^d^ College of Agriculture, Nanjing Agricultural University, Nanjing 210095, China

^e^ Department of Soil Science of Temperate Ecosystems, Department of Agricultural Soil Science, Georg August University of Göttingen, Göttingen 37075, Germany

^f^ Peoples Friendship University of Russia (RUDN University), Moscow 117198, Russia

^g^ CIRAD, UPR HORTSYS, Montpellier, France

^h^ Institute of Biological and Environmental Sciences, University of Aberdeen, Cruickshank Building, St Machar Drive, Aberdeen AB24 3UU, UK

*These authors contributed equally

**Corresponding authors:

Huadong Zang ([zanghuadong@cau.edu.cn](mailto:zanghuadong@cau.edu.cn))

**Supplementary Table 1** **The average annual belowground net C input for the world’s top 50 wheat, maize, and rice producing countries.** The required yield data for these calculations were sourced from the latest production statistics (2023) provided by the Food and Agriculture Organization (FAO) (https://www.fao.org).

| **Countries** | **Wheat** | | **Countries** | **Maize** | | **Countries** | **Rice** | |
| --- | --- | --- | --- | --- | --- | --- | --- | --- |
|  | **Tg yr^-1^** | **Mg ha^-1^ yr^-1^** |  | **Tg yr^-1^** | **Mg ha^-1^ yr^-1^** |  | **Tg yr^-1^** | **Mg ha^-1^ yr^-1^** |
| **Total average** | **2.70** |  | **Total average** | **1.76** |  | **Total average** | **2.38** |  |
| China | 56.41 | 2.39 | United States | 90.41 | 2.58 | China | 71.78 | 2.46 |
| India | 45.66 | 1.45 | China | 67.07 | 1.52 | India | 71.32 | 1.49 |
| Russian | 37.79 | 1.31 | Brazil | 30.61 | 1.37 | Bangladesh | 20.22 | 1.74 |
| United States | 20.37 | 1.35 | Argentina | 9.61 | 1.19 | Indonesia | 18.62 | 1.82 |
| Australia | 17.02 | 1.32 | India | 8.84 | 0.82 | Vietnam | 15.01 | 2.11 |
| France | 14.87 | 2.97 | Ukraine | 7.20 | 1.81 | Thailand | 11.41 | 1.02 |
| Canada | 13.20 | 1.24 | Mexico | 6.39 | 0.99 | Myanmar | 8.85 | 1.37 |
| Pakistan | 11.63 | 1.29 | Indonesia | 4.64 | 1.87 | Philippines | 6.92 | 1.44 |
| Türkiye | 9.09 | 1.33 | Russian | 3.85 | 1.60 | Pakistan | 5.11 | 1.40 |
| Ukraine | 8.93 | 1.92 | South Africa | 3.81 | 1.47 | Cambodia | 4.45 | 1.24 |
| Germany | 8.89 | 3.07 | Canada | 3.50 | 2.30 | Brazil | 3.55 | 2.39 |
| Iran | 5.78 | 0.93 | France | 2.98 | 2.26 | Japan | 3.50 | 2.37 |
| United Kingdom | 5.77 | 3.36 | Nigeria | 2.56 | 0.45 | United States | 3.42 | 2.96 |
| Poland | 5.34 | 2.18 | Ethiopia | 2.32 | 0.91 | Nigeria | 3.07 | 0.68 |
| Argentina | 5.19 | 0.94 | Pakistan | 2.28 | 1.39 | Nepal | 1.97 | 1.37 |
| Kazakhstan | 5.00 | 0.38 | Türkiye | 2.09 | 2.18 | Egypt | 1.93 | 3.01 |
| Egypt | 4.01 | 2.97 | Poland | 2.08 | 1.66 | Madagascar | 1.77 | 0.85 |
| Romania | 3.97 | 1.71 | Romania | 2.03 | 0.92 | South Korea | 1.69 | 2.39 |
| Brazil | 3.19 | 0.96 | Philippines | 1.95 | 0.77 | Sri Lanka | 1.56 | 1.34 |
| Italy | 2.85 | 1.52 | Tanzania | 1.86 | 0.44 | Laos | 1.32 | 1.51 |
| Bulgaria | 2.83 | 2.32 | Egypt | 1.65 | 1.74 | Iran | 1.24 | 1.66 |
| Uzbekistan | 2.67 | 2.17 | Serbia | 1.54 | 1.67 | Tanzania | 1.24 | 1.03 |
| Hungary | 2.45 | 2.33 | Hungary | 1.45 | 1.88 | Guinea | 1.22 | 0.52 |
| Ethiopia | 2.40 | 1.29 | Italy | 1.24 | 2.49 | Peru | 1.16 | 2.79 |
| Czechia | 2.17 | 2.66 | Thailand | 1.15 | 1.08 | Mali | 1.04 | 1.15 |
| Lithuania | 1.84 | 1.96 | Bangladesh | 1.07 | 2.14 | Colombia | 1.04 | 1.55 |
| Afghanistan | 1.78 | 0.89 | Germany | 1.04 | 2.24 | Malaysia | 0.75 | 1.22 |
| Iraq | 1.75 | 1.05 | Paraguay | 1.04 | 1.18 | Côte d'Ivoire | 0.69 | 1.01 |
| Morocco | 1.72 | 0.71 | Vietnam | 1.03 | 1.17 | Congo | 0.63 | 0.33 |
| Spain | 1.67 | 0.85 | Kenya | 0.99 | 0.41 | North Korea | 0.61 | 1.59 |
| Denmark | 1.47 | 3.00 | Ghana | 0.84 | 0.65 | Senegal | 0.52 | 1.32 |
| Mexico | 1.44 | 2.56 | Malawi | 0.81 | 0.45 | Ghana | 0.50 | 1.36 |
| Serbia | 1.42 | 2.09 | Mali | 0.79 | 0.52 | Sierra Leone | 0.48 | 0.53 |
| Syrian | 1.27 | 0.95 | Zambia | 0.76 | 0.53 | Italy | 0.48 | 2.26 |
| Sweden | 1.14 | 2.25 | Angola | 0.75 | 0.27 | Uruguay | 0.47 | 3.22 |
| Algeria | 1.03 | 0.57 | Nepal | 0.69 | 0.73 | Ecuador | 0.45 | 1.32 |
| Slovakia | 1.03 | 2.53 | Spain | 0.66 | 2.74 | Argentina | 0.40 | 2.32 |
| Belarus | 0.99 | 1.49 | Uganda | 0.65 | 0.59 | Russian | 0.37 | 1.98 |
| Latvia | 0.88 | 1.68 | Bulgaria | 0.57 | 1.06 | Guyana | 0.34 | 2.11 |
| Nepal | 0.87 | 1.24 | Congo | 0.54 | 0.18 | Paraguay | 0.34 | 1.98 |
| South Africa | 0.85 | 1.57 | North Korea | 0.53 | 0.91 | Dominican Republic | 0.33 | 1.74 |
| Azerbaijan | 0.74 | 1.36 | Cameroon | 0.51 | 0.43 | Türkiye | 0.31 | 2.77 |
| Belgium | 0.74 | 3.48 | Myanmar | 0.51 | 0.90 | Venezuela | 0.25 | 1.29 |
| Austria | 0.72 | 2.56 | Mozambique | 0.49 | 0.18 | Bolivia | 0.24 | 1.16 |
| Moldova | 0.64 | 1.71 | Austria | 0.49 | 2.30 | Afghanistan | 0.21 | 1.60 |
| Uruguay | 0.55 | 2.08 | Guatemala | 0.48 | 0.42 | Burkina Faso | 0.17 | 0.82 |
| Greece | 0.54 | 1.21 | Benin | 0.48 | 0.23 | Nicaragua | 0.17 | 2.08 |
| Chile | 0.53 | 2.46 | Burkina Faso | 0.46 | 0.38 | Australia | 0.17 | 3.28 |
| Paraguay | 0.53 | 1.19 | Croatia | 0.46 | 1.72 | Benin | 0.17 | 1.29 |

**Supplementary Table 2** **Details of the publication search in this study.**

| **Database** | **Search strings** | **Search strings** | **Publications included** |
| --- | --- | --- | --- |
| Web of Science Core collection  (http://www.webofknowledge.com) | TS=("crop isotope labeling*" OR "C allocation*" OR "labeling and crop rhizodeposition*" OR "labeling and crop photosynthesized C") | Publication years: 1986-2024  Document Types: Article  Languages: English | 131 articles |
| China National  Knowledge Infrastructure (https://www.cnki.net) | TKA = '光合碳分配 (photosynthesized C allocation)' + '作物同位素标记 (crop isotope labelling)' + '碳分配 (C allocation)' + '同位素标记和作物根际沉积 (labeling and crop rhizodeposition)' + '同位素标记和光合同化碳 (labeling and crop photosynthesized C)' | Publication years: 1986-2024  Document Types: Article  Languages: Chinese | 32 articles |

**Supplementary Table 3** **The performance of random forest and gradient boosting machine models in estimating belowground C input.** We analysised net rhizodeposition and net C input using 746 observations collected from 1986 to 2024. RF: Random forest; GBM: Gradient boosting machine; MSE: Mean Squared Error; RMSE: Root Mean Square Error; MAD: Mean Absolute Deviation.

| Paramenters | Net rhizodeposition | | Net C input | |
| --- | --- | --- | --- | --- |
|  | RF | GBM | RF | GBM |
| MSE (%^2^) | 17.87 | 7.08 | 28.85 | 7.76 |
| RMSE (%) | 4.23 | 2.66 | 5.37 | 2.79 |
| MAD (%) | 1.43 | 0.85 | 3.19 | 0.96 |
| R^2^ | 0.69 | 0.88 | 0.77 | 0.94 |

**Supplementary Fig. 1** **Global distribution of study sites.** The dataset covered 746 observations for belowground C input from 101 studies from 1986 to 2024. Red dots referred to the experimental sites. The map was created with QGIS version 3.32 (Open Source Geospatial Foundation Project, http://qgis.osgeo.org). The global soil organic carbon (SOC) stock data (0-30 cm) was sourced from the FAO Soils Portal (<https://www.fao.org/home/en/>). The global map was downloaded from natural earth (<http://www.naturalearthdata.com/>).

**Supplementary Fig. 2 Plant photosynthesized C allocation in various pools.** Boxplots displayed the data distribution of shoot respiration, shoot-C, root-C, net rhizodeposition, and root-derived CO_2_ based on 746 observations. The solid black circles represented the mean values.

**Supplementary Fig. 3 Net rhizodeposition under different root separation methods.** (a) Net rhizodeposition is expressed as a proportion of total assimilated ^13^C/^14^C. (b) Net rhizodeposition as a proportion of net C input. Four protocols were compared: fine sieving (≤ 0.6 mm mesh), coarse sieving (> 0.6 mm mesh), water washed, and manual hand-picking. The diamonds with error bars indicate mean values with 95% confidence intervals. Different lowercase letters indicate significant differences (*p* < 0.05). Sample sizes are marked below each group.

**Supplementary Fig. 4 Quantification of** **photosynthesized C allocation in the plant-soil system for various crops.** Overview of photosynthesized C allocation into shoot respiration, shoot-C, root-C, net rhizodeposition, and root-derived CO_2_ for barley (a), wheat (b), maize (c), rice (d), legumes (e), and grasses (f). The percentage values indicated the averages of all collected data for each crop after standardization. The absolute values (kg C ha^-1^ year^-1^) of C partitioning were shown in brackets and calculated based on the gross primary production (GPP) of each crop. The GPP data of barley, wheat, maize, rice, and legumes were calculated based on Bolinder et al. (2007), and grasslands based on Riederer et al. (2015).

**Supplementary Fig. 5 PRISMA diagram showing the process of locating publications included in the present analysis.**

**Supplementary Fig. 6** **Plant photosynthesized C allocation in shoot respiration and root-derived CO_2_ depending on the chase time.** Shoot respiration and root-derived CO_2_ showed as percentage of total assimilated ^13/14^C. An exponential function (y = a × (1 − exp^(-b × time)^) was fitted to all data points for shoot respiration and root-derived CO_2_. The gray area represented the 95 % confidence band.

**Supplementary Fig. 7 Factors determining plant net rhizodeposition (a-c) and net C input (d-f) with different data sets.** The meta-forest models were fitted with different numbers of variables by removing the less important variables containing missing values. a, 12 variables, n = 175; b, 11 variables (no variable “MAP”), n = 175; c, 10 variables (no variable “silt”), n = 315 for net rhizodeposition. d, 12 variables, n = 175; e, 11 variables (no variable “MAT”), n = 191; f, 10 variables (no variable “silt”), n = 331 for net C input. Crop types included barley, wheat, maize, rice, legumes, grasses, and other crops. Methods included pulse and continuous labeling. Types included field and control condition. The ecosystems included agroecosystem and grass ecosystem.

**Supplementary Fig. 8 Factors determining plant belowground C input.** The relative importance of the variables in explaining the net rhizodeposition (a, b) and net C input (c, d) was assessed based on random forest and gradient boosting machine models. Bar charts indicate the relative importance using root mean square error loss after permutation. The crop types included barley, wheat, maize, rice, legumes, grasses and, other crops. The ecosystems included croplands and grasslands. The detailed performance of random forest and gradient boosting machine models can be found in Supplementary Table 2.

**Supplementary Fig. 9 Data distribution of belowground C input depending on various factors.** Scatter plots showed individual data of net rhizodeposition and net C input with soil organic C, plant age, and chase time using 746 observations collected from 1986 to 2024.

**Supplementary Fig. 10 The upper and lower limit of global belowground net C input.** Global map of annual belowground net C input from wheat (a, b), maize (c, d), and rice (e, f). The lower and upper values were shown in left (a, c, e) and right side (b, d, f), respectively. The gray areas represented no value. The global yield data for the three crops (wheat, maize, and rice) were obtained from Iizumi and Sakai (2020). The smallest and largest percentage values for net C input (generalized) for the three crops were calculated as averages based on all collected data for each crop, representing a percentage of the gross primary production (GPP). According to the relationship between crop yield and GPP based on Bolinder et al. (2007), we mapped the global spatial distribution of belowground net C input with QGIS version 3.32 (Open Source Geospatial Foundation Project, <http://qgis.osgeo.org>). The global map was downloaded from Natural Earth (<http://www.naturalearthdata.com/>).
